# How fast could HIV change gene frequencies in the human population?

**DOI:** 10.1098/rspb.2009.2073

**Published:** 2010-03-10

**Authors:** Deborah Cromer, Steven M. Wolinsky, Angela R. McLean

**Affiliations:** 1 Zoology Department, Institute for Emerging Infections, James Martin 21st Century School, University of Oxford, Oxford OX1 3PS, UK; 2 Division of Infectious Diseases, Northwestern University Feinberg School of Medicine, Chicago, IL 60611, USA

**Keywords:** genetic selection, HIV, mathematical modelling, population genetics

## Abstract

Infectious diseases have the potential to act as strong forces for genetic selection on the populations they affect. Human immunodeficiency virus (HIV) is a prime candidate to impose such genetic selection owing to the vast number of people it infects and the varying susceptibility of different human leucocyte antigen (HLA) types to HIV disease progression. We have constructed a model of HIV infection that differentiates between these HLA types, and have used reported estimates of the number of people infected with HIV and the different rates of progression to acquired immunodeficiency syndrome (AIDS) to provide a lower bound estimate on the length of time it would take for HIV to impose major genetic change in humans. We find that an HIV infection similar to that currently affecting sub-Saharan Africa could not yet have caused more than a 3 per cent decrease in the proportion of individuals who progress quickly to disease. Such an infection is unlikely to cause major genetic change (defined as a decrease in the proportion of quickly progressing individuals to under 50 per cent of their starting proportion) until 400 years have passed since HIV emergence. However, in very severely affected populations, there is a chance of observing such major genetic changes after another 50 years.

## 1. Introduction

Human immunodeficiency virus (HIV) has had a dramatic effect on population age structure and life expectancy in the developing world (Pope & Haase 2003). In 2007, there were a recorded 33 million people infected, and this number is rising by around 2.7 million each year (UNAIDS 2008). In the absence of anti-retroviral drugs, progression to acquired immunodeficiency syndrome (AIDS), followed shortly by death, occurs after an average of 11 years (Munoz *et al*. 1989). Genetic variability of the host has been shown to have some effect on the rate of HIV disease progression as well as on HIV resistance to infection. Potential therefore exists for the virus to exert selective pressure on its human host, just as the host has applied selective pressure to the virus (Heeney *et al*. 2006; Kawashima *et al*. 2009). This paper asks how fast, and to what degree, HIV might alter the genetic structure of the human population.

**Figure 1.**
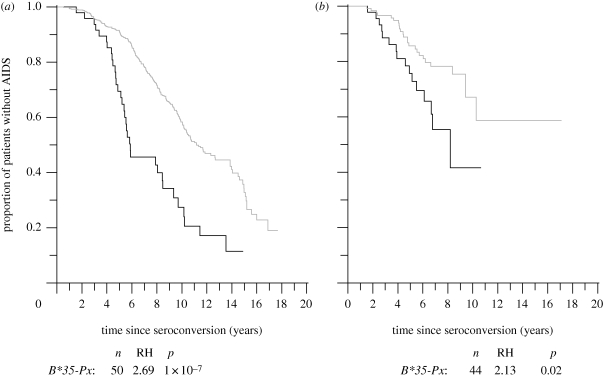
Kaplan Meyer curves showing the effect of the HLA-B*35-Px and HLA-B*53 alleles on survival for (*a*) white (*n* = 510) and (*b*) black (*n* = 181) HIV-infected individuals. Grey line, no B*35/53; black line, B*35-Px heterozygotes. Figure is adapted from fig. 2 of Gao *et al.* (2001).

A relationship between human leucocyte antigen (HLA) type and progression towards AIDS and death has been shown in a number of studies. Individuals homozygous at one or more HLA class I loci progress to AIDS faster than those who are heterozygous (relative hazard of progression ≥ 1.68; Carrington *et al*. 1999). Certain HLA alleles, such as HLA-B*57 and HLA-B*27, are associated with slower progression to AIDS (O'Brien *et al*. 2001; Carrington & O'Brien 2003). Other HLA alleles, HLA-B*35-Px and HLA-B*53, have been linked to faster HIV disease progression and AIDS-defining illness (Carrington *et al*. 1999; Gao *et al*. 2001). The HLA-B*35-Px and HLA-B*53 alleles have a frequency of 4.4 per cent in the white population and 12.8 per cent in the black population (Gao *et al*. 2001). The effect of the HLA-B*35-Px/B*53 subtype on progression to AIDS is significant, and is shown in figure 1. In a large cohort study, more than 50 per cent of white subjects possessing the HLA-B*35-Px or HLA-B*53 allele progressed to AIDS within 6 years, while it took over 11 years for this to occur in a similar percentage of subjects not possessing the alleles. Time frames in black subjects were 8 years in those with the alleles and over 17 years in those without (Gao *et al*. 2001). Thus, the median time to AIDS in individuals possessing the HLA-B*35-Px or HLA-B*53 genotype is approximately half that in the rest of the population. As well as this direct evidence for an association between HLA genotype and progression to AIDS, indirect evidence has been found in the form of relationships between HLA supertype and viral load (Trachtenberg *et al*. 2003), and between viral load and progression to AIDS and death (Mellors *et al*. 1996).

Infectious diseases have the ability to select for and against certain genotypes in an infected population. An obvious example of this is the selection in parts of Africa for the mutation responsible for sickle cell anaemia. This mutation is protective against infection with *Plasmodium falciparum* (one of the common strains of malaria; Allison 1954), and therefore natural selection for it has occurred over thousands of years (Currat *et al*. 2002). Similarly, it has been suggested that the *CCR5*-Δ*32* deletion allele that is protective against HIV has been previously selected for in Europe by an infectious disease (Stephens *et al*. 1998; Galvani & Slatkin 2003).

The speed of genetic selection is strongly correlated with the prevalence of a disease. Assuming that other disease parameters are equal (e.g. probability of transmission, death rate from disease), an infectious disease with high prevalence will exert stronger selective pressure on the population than one with lower prevalence. A significant factor affecting disease prevalence is the changing behaviour in response to the HIV epidemic that has occurred in many populations (Martin 1987; Nelson *et al*. 1996; Stoneburner & Low-Beer 2004). Evidence of a decrease in the number of people having sex with a non-regular partner and an increase in condom use has been seen in a number of countries in Africa (Stoneburner & Low-Beer 2004; UNAIDS 2005; Cheluget *et al*. 2006) and has contributed to a decline in HIV prevalence in these countries.

The selective pressure of HIV on the genetic makeup of the human population is currently unknown. It is likely that prolonged exposure to HIV at high levels of infection could cause selection for protective HLA alleles and against alleles that speed progression towards AIDS and death. Given the severity of the HIV epidemic, it is possible that the virus has already exerted a strong selective pressure, and so altered the ratios of certain alleles within the human population. In this paper, we analyse whether, given the known growth of HIV, it is possible that HIV has already caused significant selection against HLA types that enhance disease progression and death.

## 2. Modelling approach

To find an estimate for how quickly HIV could remove a certain allele from the human population, we assumed that individuals possessing such an allele progress to AIDS (and subsequently death) at a significantly faster rate than those without the allele. We set up a model of HIV infection that includes diploid population genetics involving such a frail allele (*f*) and a normal allele (*n*). We allow ‘frailty’ to be partially dominant in the main section of the paper, and consider dominant and recessive modes of inheritance in the electronic supplementary material. We consider frequency changes of the frail and normal alleles within the population owing to the disease, although frequency-dependent selection itself does not play a role in the model. We also develop a ‘back of an envelope’ calculation that links prevalence levels to selective pressure and so allows simple calculations of the expected impact of a given prevalence.

The equations we have used to model HIV transmission dynamics (equation (4.1*a*,*b*)) are based on those presented by Anderson *et al*. (1988). We have extended the model to include three different groups in the population—individuals homozygous for the normal allele, heterozygotes and individuals homozygous for the frail allele. When modelling the dynamics of an infectious disease in a single intermixing population, the initial rate of increase and the eventual plateau in the number of infected individuals are inextricably intertwined. It takes hundreds of years to achieve a plateau level of 30 per cent disease prevalence in the population. What we have seen with HIV in the worst-affected populations is a rapid rise to around 30 per cent of the population infected after several decades, and a plateau at this level. It is not possible to achieve such dynamics from a model of a population with homogeneous, time-stable rates of infection probability and partner change even by introducing a frail allele. Thus, we altered the model so that it was capable of reflecting observed HIV dynamics by allowing the probability of infection and the rate of partner change to vary with time. These parameter changes capture the behaviour changes (and thus lower rates of infection) that have been observed in many parts of Africa, and ensure that our model can produce dynamics that occur over the observed time scales of HIV spread in the population.

## 3. Prevalence levels

The HIV epidemic is taking a number of different courses throughout Africa (and indeed throughout the world). Reports from some parts of Africa show that the epidemic is already declining in prevalence (Stoneburner & Low-Beer 2004; UNAIDS 2005; Cheluget *et al*. 2006), while other reports show that prevalence has continued to increase and recently appears to have plateaued (in some areas at levels as high as 30 to 40 per cent; Asamoah-Odei *et al*. 2004). The prevalence of HIV is a key determinant of its selective pressure. From available prevalence data, we selected three sites to represent distinct courses the epidemic can take. We will discuss the effect that each of these three prevalence ‘scenarios’ could have on the genetic makeup of the population.

**Figure 2.**
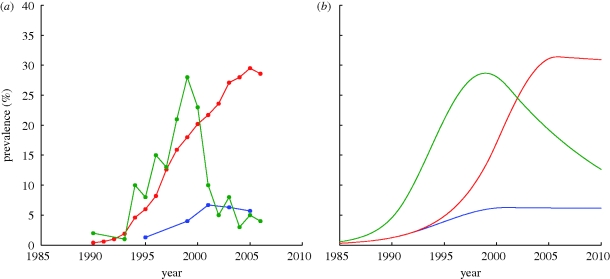
Examples of different prevalence patterns taken by HIV. (*a*) Data from three different sites: blue line, low (Jalingo, Nigeria); red line, high (Eastern Cape Province, South Africa); green line, disappearing (Meru, Kenya). (*b*) Model prevalence outputs from three parameter sets for partial dominance with 10 per cent of the population initially heterozygous for the frail allele: blue line, low simulated; red line, high simulated; green line, disappearing simulated. Parameters used are *ν* = 0.04, *μ* = 0.02, *α*_1_ = 0.071, *α*_2_ = 0.162, *α*_3_ = 0.313, *ε* = 0.7, *y*_0_ = 0.1%. Parameters for equation (4.2) vary for the different prevalence scenarios, and are: low prevalence, *φ*_0_ = 0.4, *φ*_end_ = 0.117, *τ*_2_ = 10, *τ*_2_ = 20; high prevalence, *φ*_0_ = 0.4, *φ*_end_ = 0.124, *τ*_2_ = 19, *τ*_2_ = 25; disappearing prevalence, *φ*_0_ = 0.55, *φ*_end_ = 0.02, *τ*_2_ = 10, *τ*_2_ = 20.

The selected prevalence scenarios are shown in figure 2*a*. We label them as (i) low, (ii) high and (iii) disappearing. ‘Low’ means an infection that rises to infect 6 per cent of the population and plateaus at this level. An example of such an infection is found in Jalingo, Nigeria (WHO 2008*a*). ‘High’ means an infection that rises to infect around 30 per cent of the population, and plateaus at this level. An example of such an infection is found in the Eastern Cape Province of South Africa (WHO 2008*b*). ‘Disappearing’ means an infection that rises quickly to infect close to 30 per cent of the population, but then decreases after this peak. An example of such an infection is found in Meru, Kenya (National AIDS Control Council & National AIDS and STD Control Programme 2007). We select parameter values for the model that allow us to produce prevalence curves similar to those in figure 2*a*. The prevalence curves produced by the model are shown in figure 2*b*.

## 4. The model

When defining the model, we use subscripts of *i* to denote the different genotypes within the population. The subscript *i* = 1 refers to the subset of the population homozygous for the normal allele (*nn*), *i* = 2 refers to the heterozygous subset (*nf*) and *i* = 3 refers to the subset that are homozygous for the frail allele (*ff*). The susceptible population in subset *i* is represented by *X*_*i*_(*t*) and the infected population by *Y*_*i*_(*t*). The total population in subset *i* is represented by *N*_*i*_(*t*), with *N*_*i*_(*t*) = *X*_*i*_(*t*) + *Y*_*i*_(*t*). Note that although *X*_*i*_(*t*), *Y*_*i*_(*t*) and *N*_*i*_(*t*) are functions of time, we have omitted to explicitly state this where it simplifies notation. The subscript of ‘tot’ indicates a sum over all values of *i*. So for example, 
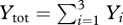
. The terms *B*_*Xi*_ and *B*_*Yi*_ define the children born to infected and uninfected mothers, respectively, in group *i*, and are defined in equations (S1.1*a*–*f*) in the electronic supplementary material.

The model we use is
4.1a
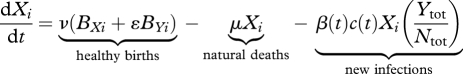

and
4.1b
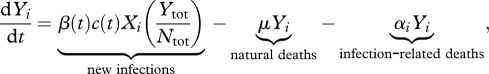

where ν gives the birth rate in the healthy population, *μ* is the rate of death from non-infection-related causes and *ε* is the fraction of uninfected offspring born to infected mothers. This means that (1 − *ε*) is the fraction of offspring who acquire infection through perinatal transmission. The first part of the healthy births term in equation (4.1*a*) represents the children born to uninfected mothers, and the second part represents the children born to infected mothers but who do not acquire the disease through perinatal transmission. We do not include the children who acquire disease through perinatal transmission in our model, as these children are likely to die at a young age from disease (prior to reproductive age) and so will not contribute significantly to population dynamics. Omitting these children from the model does not alter the results. We assume that all children born to healthy mothers are healthy.

Within the new infections term (final term of equation (4.1*a*) and first term of equation (4.1*b*)), the parameter *β*(*t*)*c*(*t*) is a product of *β*(*t*), the probability of acquiring HIV from one infected partner at time *t*, and *c*(*t*), the average rate of acquiring partners at time *t*. Changes in practices such as increased condom use will cause *β*(*t*) to decrease over time. Similarly, changes in the number of non-regular sexual partners and rates of partner acquisition will cause *c*(*t*) to decrease. We allow the combined function *β*(*t*)*c*(*t*) to decrease over time in order to capture these behaviour changes. The combined function *β*(*t*)*c*(*t*) starts from an initial value, *φ*_0_, and decreases to a lower value, *φ*_end_, over a time interval from *τ*_1_ to *τ*_2_, and takes the following form:

4.2
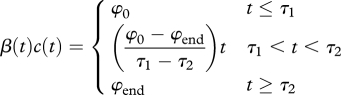


The values used for *β*(*t*)*c*(*t*) are given in the legend of figure 2 and table S2 in the electronic supplementary material, and are in agreement with reported estimates of behaviour change (Stoneburner & Low-Beer 2004; Cheluget *et al*. 2006); however, it is important to note that model results are dependent only on the prevalence of HIV predicted by the model, which we have matched to observed HIV prevalence levels (figure 2), and do not depend on the exact parameter values chosen. Indeed, different parameter values that give rise to the same prevalence levels will yield identical results (see the electronic supplementary material).

In this model, we have assumed that random mating occurs between infected and uninfected individuals, and that the population has a uniform age structure. Allowing for assortative mating or including age structure in the population would increase the length of time required for selection against the frail allele to occur (see the electronic supplementary material).

### (a) Implementing the model

The parameters used to implement the model are given in the legend of figure 2, and in tables S1 and S2 in the electronic supplementary material. Results of modelling a low-prevalence infection, a high-prevalence infection and a disappearing infection are shown in figure S1 in the electronic supplementary material. For all diploid simulations, we have assumed that frailty is partially dominant and that initially 10 per cent of the population is heterozygous for the frail allele. The results of simulations with different starting ratios of frail individuals are discussed in the electronic supplementary material. These assumptions agree with data from Gao *et al*. (2001), which suggest partial dominance and a 4 to 13 per cent prevalence of the B*35-Px/B*53 allele. In this and all simulations, we assume that the genetic makeup of the population is initially in Hardy–Weinberg equilibrium. This equilibrium determines that initially 0.3 per cent of the population is homozygous for the frail allele. In all cases, the prevalence of the frail allele decreases and the prevalence of the normal allele increases in the population. This results in a decrease in the percentage of individuals with the frail phenotype in the population.

## 5. Time until genetic modification of the population during an HIV epidemic

**Figure 3.**
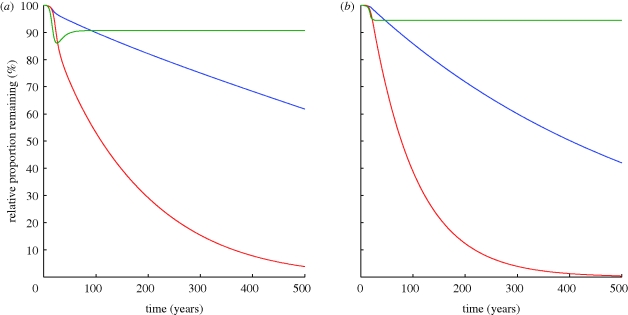
Relative decreases in frequencies of the frail allele and phenotype in the years following an HIV epidemic. Proportions are shown relative to the initial proportion of frail alleles within the population. (*a*) Relative frequency decreases of the frail allele obtained when solving equation (4.1*a*,*b*) in the presence of a low-prevalence (blue line), high-prevalence (red line) and disappearing (green line) infection. We have assumed that inheritance of the frail allele is partially dominant and that initially 10 per cent of the population is heterozygous for the frail allele. Parameters are as for figure 2*b*. (*b*) Relative frequency decreases in the proportion of frail susceptible individuals estimated using equation (6.5). ν = 0.04, *ε* = 0.7 and *g*(*t*) taken from figure 2*a*. Results are shown for a simplified low-prevalence (blue line), simplified high-prevalence (red line) and simplified disappearing (green line) infection.

To analyse the effect of the current HIV epidemic on the genetic makeup of the population, we consider the genetic changes during the high, low and disappearing epidemics shown in figure 2. For the first part of our analysis, we make the same assumptions as above, namely that frailty is partially dominant, that 10 per cent of the population is initially heterozygous for the frail allele and 0.3 per cent is initially homozygous for the allele. This implies an initial prevalence of the frail allele of 5.3 per cent. We allow frail individuals to proceed to death in 5.5 years, half the time for normal individuals, who die 11 years after becoming infected. Those who are homozygous for the frail allele die even faster, in 3 years. These timings are inferred from figure 1 and Gao *et al*. (2001). Figure 3*a* shows the relative proportion of the frail allele remaining in the population compared with its starting proportion predicted by our model for each of the three prevalence scenarios given in figure 2.

For a low-prevalence infection, the frequency of the frail allele is predicted to remain above 90 per cent of its starting proportion until at least 2080 and only to decrease to 62 per cent of its starting proportion after 500 years. For a high-prevalence infection, it would take 110 years for the frail allele to drop below 50 per cent of its initial proportion and 260 years for it to drop below 20 per cent of its initial ratio. It should be noted that for this to occur, the infection prevalence is required to remain at 30 per cent for the next few hundred years. If the prevalence of HIV were to decline, these estimates would increase. We would expect the frail allele frequency in the population to be above 72 per cent of its starting proportion for the next 20 years, indicating that selection is still quite slow. During an infection that peaks and disappears (green lines in figure 3*a*), a maximum decrease in the proportion of the frail allele is attained following the peak of infection when it drops to 86 per cent of its starting value. The long-term proportion of the frail allele is 91 per cent of that before the epidemic.

We can calculate the expected decrease in the percentage of the frail alleles present over the 30 years that HIV has affected population dynamics. We find that for a low prevalence infection, the proportion of frail alleles has decreased by just 4 per cent to 96 per cent of its initial proportion. For a high-prevalence infection, it has decreased to 83 per cent of its starting proportion; and for a disappearing infection to 87 per cent of its starting proportion.

We observe that an infection that peaks and disappears does not dramatically alter allele and phenotype frequencies (green line in figure 3*a*), as it does not impose lasting selection on the population. Therefore, for the remainder of our analysis, we focus on the low- and high-prevalence infection scenarios, as it is these scenarios that have the potential to result in sustained genetic selection.

## 6. A simplified model to provide a lower bound estimate

We next attempt to provide a lower bound estimate on the time it would take for HIV to impose genetic selection on the human population owing to selection against a frail genotype. Our aim in providing such a lower bound estimate is to provide a generalized result that is not dependent on as many parameters and removes some of the assumptions about the disease, while still being able to provide insight into the maximum speed at which HIV is capable of imposing genetic selection.

We wish to find a lower bound estimate on how quickly HIV could remove some genes from the human population, so we assume a very dramatic impact on a small subset. We make three additional simplifying assumptions in this model. These are as follows:

— Within the frail subset, there is 100 per cent failure to reproduce healthy children, as infected individuals either pass on the disease through perinatal transmission or die prior to reproduction.
— The ‘gene’ for frailty or normality is passed on directly from mother to child and the father's genotype has no effect.
— Only uninfected individuals are considered. Infected individuals die at a significantly faster rate than uninfected individuals, and therefore are removed from the population relatively quickly after becoming infected. They thus have a less significant effect on the population structure, and removing them from the analysis should not greatly alter the results.

These are all assumptions that will tend to speed up the rate of genetic change, while greatly simplifying the model so that analytical results are possible. The simplified model will therefore provide a conservative estimate of the time taken for genetic change to occur.

### (a) Setting up the simplified model

In this section, we use the subscripts of *j* to denote the different phenotypes within the population. The subscript *j* = 1 refers to the normal phenotype and the subscript *j* = 2 refers to the frail phenotype. As previously, the susceptible population in subset *j* is represented by *X*_*j*_(*t*) and the infected population by *Y*_*j*_(*t*).

The model we use is
6.1a
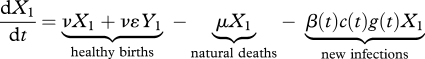

and
6.1b
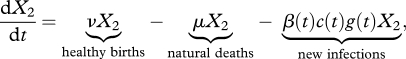

where ν, *μ*, *β*(*t*), *c*(*t*) and *ε* are defined as in §5 and *g*(*t*) is a function representing the prevalence of HIV in the entire population at time *t*, such that 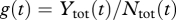.

### (b) Solutions to the simplified model

To solve equation (6.1*a*), we observe that *Y*_1_ = *Y*_1_/*X*_1_ *X*_1_ ≈ *Y*_tot_/*X*_tot_ *X*_1_ as the normal subset of the population is much larger than the frail subset. Therefore, we can write 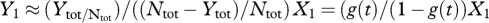 and we have
6.2a
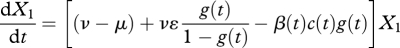

and
6.2b
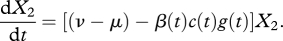



We can then find solutions to equations (6.2*a*,*b*) as
6.3a
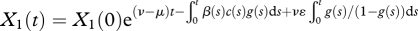

and
6.3b
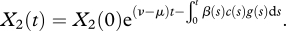



Equations (6.3*a*,*b*) can be combined to give

6.4
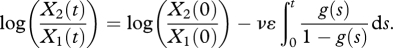


We let 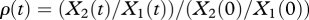 be the change in the ratio of frail to normal susceptible individuals compared with the starting ratio (i.e. the relative change in the proportion of frail susceptible individuals), and we have

6.5
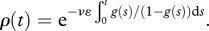


This allows us to determine a conservative estimate for the relative decrease in the proportion of frail susceptible individuals using only known prevalence levels, the birth rate and the rate of perinatal transmission. Analytical expressions for *ρ*(*t*) and the related function *T*(*ρ*), the time taken for the ratio of frail to normal susceptible individuals to drop below *ρ* per cent of its starting ratio, can be determined when the prevalence function, *g*(*t*), takes certain simple forms. Expressions for ‘broken-stick’ and triangular functions of *g*(*t*) are given in the electronic supplementary material.

A further simplification can be obtained by assuming that the percentage of infected individuals in the population has been constant since HIV was first detected (i.e. HIV suddenly appeared and infected *γ* per cent of the population). Then, we have *g*(*t*) = *γ*, and equation (6.4) takes the form

6.6
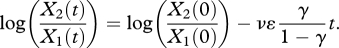


The selection coefficient, *s*, against the frail subset is given by
6.7
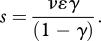

This is also the difference between the Malthusian parameters of the frail and normal phenotypes that is obtained when substituting *g*(*t*) = γ into equations (6.3*a*,*b*), and is therefore the selection coefficient that is obtained using standard population genetics results (Dykhuizen & Hartl 1980; Hartl & Clark 1997). We can use the selection coefficient to determine an expression for *T*(*ρ*), the time taken for the ratio of frail to normal susceptible individuals to drop below *ρ* per cent of its starting ratio, as

6.8
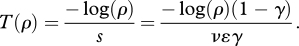


For an infection with 6 per cent prevalence (low) we have *s* = 0.00179, and for a high prevalence infection (30 per cent prevalence) we have *s* = 0.012.

### (c) Time until genetic modification of the population using the simplified model

In figure 3*b*, we show graphs of *ρ*(*t*), the relative decrease in the proportion of frail susceptible individuals, calculated using equation (6.5) for each of the three prevalence scenarios of figure 2*a*. We have allowed *g*(*t*) to be determined by interpolating each of the prevalence curves. For times beyond those shown in figure 2*a*, *g*(*t*) is given a constant value for the low- and high-prevalence scenarios, and is extended to a zero-prevalence infection over 4 years for the disappearing infection. The resulting curves of *ρ*(*t*) shown in figure 3*b* are significantly steeper than similarly coloured curves in figure 3*a*. This confirms that the solution to our simplified model is indeed a conservative estimate of the relative decrease in the proportion of frail individuals in the population.

These results show that even a conservative estimate of the time required for genetic selection suggests that it would take hundreds of years to significantly alter the ratio of frail to normal individuals in the susceptible population. During a low-prevalence infection, it would take over 400 years for the proportion of frails in the susceptible population to decline below 50 per cent of its starting value, and over 900 years for it to decrease below 20 per cent. Even during a high-prevalence infection, it would take over 78 years for the proportion of frails in the susceptible population to decrease to below 50 per cent of its starting value, and 158 years for it drop below 20 per cent. These times are significantly greater than the 30 years for which HIV has had an observable impact on population dynamics.

We can also calculate the maximum decrease in the ratio of frail to normal individuals in the susceptible population over the 30 years that HIV has affected population dynamics. We find that for a low-prevalence infection, this ratio could not have declined below 97 per cent of its starting value, and for a high-prevalence infection it could not have declined below 87 per cent of its initial value.

We can use equation (6.8) to calculate the time it would take for the prevalence of the frail allele in the population to decrease below *ρ* per cent of its starting proportion using the selection coefficients calculated earlier. We find that for a low-prevalence infection (*s* = 0.00179) it would take 387 years for the frail allele to decrease below 50 per cent of its starting ratio, and 899 years for the ratio to decrease below 20 per cent. During a high-prevalence infection (*s* = 0.012), it would take 58 years for a 50 per cent decrease and 134 years for the ratio to decrease below 20 per cent.

## 7. Conclusion

Our analysis suggests that although HIV has had an enormous impact on the sub-Saharan African population, it could not yet have wiped out an entire subset of this population owing to their faster progression to AIDS and death. Even an extremely conservative estimate in the most severely affected areas suggests that it would take a minimum of another 50 years (more than double the length of time for which HIV has been at a detectable level) for HIV to lower the ratio of frail individuals in the susceptible population to below 50 per cent of its ratio prior to the introduction of HIV. Our less conservative estimates suggest that it is actually likely to take more than 100 years for this to occur.

It is important to note that within our simplified model, we have made an extremely conservative assumption, namely that there is 100 per cent failure to reproduce in the frail group. This allows us to provide a lower bound estimate on the time taken for the frail subset of the population to decline. Relaxing this assumption increases numbers in the frail subset of the population and so increases the estimated time for the frail subset to disappear from the population. Even this very conservative estimate of a severe disease shows that HIV could not have already eliminated a frail subset from the population.

During this analysis, we have assumed that HIV has only had an observable impact on population dynamics since its discovery in 1981. Although HIV was around prior to this time, its prevalence was extremely low and so we have ignored this early phase. In actual fact, assuming that HIV has been around for longer than 25–30 years would have increased our estimates for the time taken for the percentage of frail individuals to decrease.

In this analysis, we have considered different long-term outcomes by analysing the effects of a low-prevalence, a high-prevalence and a disappearing-prevalence infection would have on the time required for genetic selection. However, it is probable, and indeed hopeful, that improvements in HIV drugs, a better distribution of existing drugs and the development of a prophylactic vaccine could affect our long-term predictions. The effect of each of these interventions would be to slow down the speed of genetic selection by decreasing infectivity and lowering transmission. Therefore, once again, the results presented here are estimates of the minimum time required to see significant changes in allele frequencies owing to selection imposed by HIV.

Resistance to HIV has been associated with certain genotypes that make up a small subset of the population. Individuals with a *CCR5*-Δ*32* mutation on the CCR5 chemokine receptor are highly resistant to HIV infection (Dean *et al*. 1996; Huang *et al*. 1996). In addition, an HLA supertype, A2/6802, is associated with resistance to HIV infection through both sexual and perinatal transmission (MacDonald *et al*. 2000, 2001). It has been more difficult to determine genes associated with increased susceptibility to HIV. Although some studies have suggested HLA types that may confer increased susceptibility (Cruse *et al*. 1991; Fabio *et al*. 1992; Achord *et al*. 1996; Rohowsky-Kochan *et al*. 1998; Habegger de Sorrentino *et al*. 2000), a general agreement has not been reached (Carrington & O'Brien 2003). Therefore, this work has not focused on the potential removal of a genotype from the population owing to increased susceptibility.

An issue with this work could arise if the frail subset of the population is also more susceptible to contracting HIV infection or if they have a significantly different probability of perinatal transmission. In general, however, neither the HLA types that have been associated with increased HIV susceptibility nor those that have been associated with a different probability of perinatal transmission are the same as those that have been associated with faster progression to AIDS (Rohowsky-Kochan *et al*. 1998; MacDonald *et al*. 2001; Carrington & O'Brien 2003; Farquhar & John-Stewart 2003). This means that there should be no confounding effects on our analysis from increased susceptibility or different rates of perinatal transmission. It has, however, been suggested that the HLA-B*53 allele, which causes faster progression to AIDS (Gao *et al*. 2001), may be associated with resistance to HIV (Rohowsky-Kochan *et al*. 1998). Nevertheless, if this is the case, it does not alter our conclusions as it would mean that the true time for the ratio of frail individuals to decrease below a percentage of its starting value is greater than that presented in this paper, and so the estimates presented here are once again conservative.

The results of our model are in agreement with those presented by Schliekelman *et al.* (2001), who suggested that a constant high-prevalence infection could cause a 50 per cent relative decrease in an AIDS-accelerating haplotype over a period of 100 years. This is similar to our conservative estimate of the time required for a 50 per cent relative decrease in the frail phenotype. A major advantage of our work over this previous result is that our model allows one to calculate the selection pressure of an epidemic for which the prevalence changes over time either as a result of behaviour changes or as a result of other factors.

In this model, we have assumed that male population dynamics track female population dynamics. Although there are certainly differences between male and female behaviour, this simplification has routinely been used in models of HIV dynamics (WHO & UNAIDS 2002; Brown *et al*. 2006). In order to model the male and female sections of the population separately, one would require estimates of gender-specific transmission rates and the different rates of partner formation between the sexes (Williams *et al*. 2006; Hallett *et al*. 2007). Such a model may also need to divide the population into different risk groups to capture the full range of gender-specific behaviours (Hallett *et al*. 2007; Nagelkerke *et al*. 2007). Understanding how the inclusion of these factors affects the rate of genetic selection owing to HIV would present an interesting avenue for future research.

Our analyses have not considered coevolution of host and virus. Recent data demonstrate clearly that HIV is adapting to human immune responses at the population level (Kawashima *et al*. 2009). However, in these findings, the people exerting the strongest (detected) selective pressures are members of a small subset that does particularly well. In this work, we consider the rate of disappearance of a different small subset that does particularly badly and is not a major force exerting selective pressure on the virus. In addition, any selection that is occurring in the normal or resistant section of the population is unlikely to affect the rate of disappearance of a frail subset. For the purposes of these calculations, it was therefore not necessary to include evolutionary change in HIV, or to consider selection effects in the normal population.

Despite the many conservative assumptions that are contained in our model, we have been able to conclude that a frail subset of the population, which progresses to AIDS and death more quickly than the rest of the population, could not, however, have decreased dramatically. Even in the face of an extremely severe and continuing HIV epidemic, it would take well over double the length of time for which HIV has been around for this frail subset to decrease to below half of its initial ratio in the population. This is an extremely conservative estimate, and the true length of time it would take for this subset of the population to decline is probably more than four times the duration of the current HIV epidemic.
